# Health trajectories of elderly living in French senior housing: a longitudinal perspective

**DOI:** 10.1038/s41598-023-32429-3

**Published:** 2023-04-04

**Authors:** Denis Boucaud-Maitre, Céline Meillon, Luc Letenneur, Roxane Villeneuve, Jean-François Dartigues, Hélène Amieva, Maturin Tabue-Teguo

**Affiliations:** 1 Direction de la Recherche et de l’Innovation, Centre Hospitalier le Vinatier, Bron, France; 2 Equipe EPICLIV, Université des Antilles, Fort-de-France, Martinique; 3 Univ. Bordeaux, Inserm, U1219 Bordeaux Population Health Center, Bordeaux, France; 4 Centre Hospitalo-Universitaire de Martinique, Fort-de-France, Martinique

**Keywords:** Geriatrics, Epidemiology

## Abstract

Senior housing for older adults could be an alternative or a transitional care model between home care and nursing home care. Using two longitudinal cohorts of community dwellers aged 65 years or older, we compared risks of mortality and of nursing homes admission between older adults who did or did not move to senior housing over time. In the 3C study (n = 2104, 17 years of follow-up), 143 (6.8%) participants moved into a senior housing during the follow-up. This move was associated with a lower risk of mortality (hazard ratio (HR): 0.64; 95% confidence interval (CI) 0.46–0.77) and a higher risk of nursing home admissions (HR: 1.54 (1.10–2.15)). The risks of hospitalizations (HR: 0.54 (0.40–0.73)) and falls (HR: 0.63 (0.50–0.79)) were lower. In the PAQUID study (n = 3777, 27 years of follow-up), 161 (4.3%) participants moved into a senior housing. This move was also associated with a lower mortality risk (HR: 0.72 (0.58–0.88)) and a higher risk of nursing home admissions (HR: 1.39 (1.05–1.86)). Our results showing lower risks of mortality suggest that senior housing may be a relevant model for vulnerable older adults.

## Introduction

The aging of the population in developed countries poses significant challenges for health policy worldwide. Finding the appropriate care option for dependent or vulnerable older adults remains difficult^1^. The WHO, within the framework of Decade of Healthy Ageing, promotes the integration of care and services that meet the individual needs of older people and access to long-term care^2^. To date, the most widespread social model revolves around home care and nursing home care—for people with or at risk of unmet home care needs. It has been previously reported that the majority of people aged 50 and over would like to age in their current home (85% according to a French national survey institute^3^), regardless of their functional status. Several services promote aging in place for this purpose (remote monitoring, telemedicine, home care services)^4,5^. Nonetheless, social isolation, social vulnerability, and loneliness among older people living at home^6^ increase the risk of adverse health events; they are respectively associated with a 29%, 26%, and 32% increase in mortality risks^7^. On the other hand, voluntary or involuntary nursing home admission is often the result of a deterioration in health caused mainly by falls, cognitive impairment, dependence in daily activities, or strokes^8,9^. Nursing homes are the last place of residence as well as the place of death for most institutionalized older people, after an average length of stay of three years and four months in France^10^.

The transition from one’s home to a nursing home usually disrupts residents’ social life and lifestyle^11^. Institutionalized older adults experience greater cognitive decline than community-dwelling individuals^12^, a decreased in quality of life^13^ and increased psychotropic medication use^14^ after moving into a nursing home. For these reasons, we need to study alternative and transitional care models between home care and nursing home care. Among these models, “senior housing” (also called “residential care facilities”, “independent living communities”, “assisted living facilities”, or “continual care retirement communities”) have been developed over the last decades. Usually, each resident has a private apartment and access to common areas and services. Senior housing differ in size, type (residence or village), services offered, and costs. These facilities aim to promote the autonomy of older people and help them maintain an active social life^15^. In the United States, between 980,000 and 2 million people live in these community-based residential care settings, depending on the definition we use^16^. In France, there are about 2300 residences of this type (representing approximately 120,000 accommodation places), called "Residences for autonomous seniors" accommodating an average of 48 residents each. Two-thirds are public, 30% are private non-profit and 4% are commercial. These structures have a social vocation, they receive funding and the rents are moderate. External service providers (private doctors, home nursing services, private nurses) most often provide medical care and routine care for residents. In some senior housing, there are medical staff on site, such as care assistants or nurses. Staffing levels are lower in senior housing than in nursing homes, with a staff ratio of 13 per 100 beds compared to 64 per 100 beds in nursing homes^17^. The mean age at admission is 81 years, and the mean length of stay is five years^16^. Most residents have no disability, but more than half meet the criteria for frailty (53.7%) and suffer from depressive disorders (53.5%)^18^.

Few studies have assessed the effectiveness of senior housing in preventing adverse health events (deaths or hospitalizations) compared to home care. Available studies^19–22^ suggest a higher risk of mortality in senior housing compared with home care or community. However, these studies are of short duration (1–5 years of follow-up) and the baseline medical characteristics of the older adults were not comparable between these two populations. Regarding hospitalization, one longitudinal study observed a decrease for heavy hospital use over time for older people living in senior housing compared with older people remaining at home^23^.

The choice to invest in a senior housing is an important issue for a village or a town. Thus, assessing the relevance of this investment is crucial for the community. Research is necessary to determine whether this type of geriatric facility can be an effective alternative to home care and promote higher survival rates. The present study uses data from two population-based cohorts (3C and PAQUID) of older people living at home initially and followed over time, contrary to previous studies^18–21^. Its primary objective was to compare mortality and risks of nursing home admission between older people who moved to a senior housing during follow-up and older people who did not moved into senior housing. Secondary objectives were to compare the risk of hospitalization, fall, and frailty that might explain a potential difference in survival, using data from the 3C cohort.

## Materials and methods

### The 3C study

The 3C Study is an on-going multicenter prospective cohort study conducted in France. Its primary objective was to assess the relationship between vascular factors and dementia in people aged 65 years and over. The detailed study protocol has been published elsewhere^24^. Briefly, noninstitutionalized men and women aged 65 years or over were randomly selected from electoral rolls of three French cities. The acceptance rate was 37%, resulting in 9294 participants (4931 from Dijon, 2104 from Bordeaux, and 2259 from Montpellier). Participants were followed-up every two to three years (7 follow-ups) for 17 years, even if they entered a senior housing or a nursing home. The Bordeaux sample provided information on vital status at baseline (1999–2000) and at each follow-up (n = 2104). The number of participants lost to follow-up in this sample was 277 at 17 years. By the end of the last follow-up, 1257 participants had died.

### Data from the 3C study

Trained nurses and psychologists collected the data during face-to-face interviews using standardized questionnaires. For the present study, the following variables were extracted at baseline and each follow-up: sociodemographic characteristics (gender, age, and educational level), transition to a nursing home or a senior housing, vital status, hospitalizations, falls, global cognitive performance, and functional status. The Mini-Mental State Examination (MMSE) score, ranging from 0 to 30, measured global cognitive functioning. The Instrumental Activities of Daily Living (IADL) scale (Lawton’s IADL scale) and the Activities of Daily Living (ADL) scale (Katz's scale) assessed functional status. Depression was assessed with the CES-D (Center for Epidemiologic Studies- Depression) scale. Falls were self-reported.

Frailty was assessed using the five components of the phenotype proposed by Fried et al.^25^. Weight loss was assessed in a self-reported manner (recent and unintentional weight loss of 3 kg or more or a body mass index (BMI, calculated from anthropometrical measurements) lower than 21 kg/m^2^). Participants assessed their level of exhaustion with two questions also used in the Cardiovascular Health Study: ‘‘I felt that everything I did was an effort’’ and ‘‘I could not get going.’’. Slowness was defined as the lowest quintile on a timed 6-m walking test, at usual pace, adjusted for gender and height, among participants of this study. Participants answering ‘‘yes’’ to the question ‘‘Do you have difficulty rising from a chair?’’ were categorized as frail for weakness. Low physical activity corresponded to performing daily leisure activities (such as walking, gardening, or exercising) less than once a week. For the present study, participants meeting three or more criteria were considered frail, and the others non-frail.

### Validation analysis in the PAQUID study

The PAQUID cohort started in 1988–1989 with a representative sample of 3777 participants aged 65 years and over and living at home in two French departments (Gironde and Dordogne)^26^. The selection was stratified by sex, age, and urban unit’s size. Face-to-face interviews were conducted at home every 2–3 years by specially trained neuropsychologists. Sociodemographic, environmental, and health-related data, including entry into a nursing home or a senior housing, were collected prospectively at each wave with the participant or a proxy, when self-assessment was impossible or invalid. The PAQUID program included a systematic and regular record and check of deaths with the administrative data from death certificates obtained from general practitioners and the city administrative department (date and cause of death). Data on hospitalizations, falls, and frailty were unavailable for this cohort. The number of older people lost to follow-up in this cohort was 98. By the end of the study, 3665 participants had died.

### Statistical analysis

Descriptive analyses were conducted using frequencies and percentages for categorical variables and means and standard deviations (SD) for continuous variables.

In our analyses, nursing home admission and death were considered as competing risks in a semi-parametric illness-death model accounting for left-truncation and right censoring. We used a semi-parametric approach with M-splines approximation of baseline intensities in order to obtain smooth estimates of the hazard functions^27^. This model allowed us to make predictions and obtaining the following probabilities:

- The probability of being alive without entering a nursing home.
- The probability of being alive and entering a nursing home.
- The probability of dying.

We also presented the predictions in two subgroups: people who never moved to a senior housing and people who did so during follow-up. We performed univariate and multivariate models taking age as the baseline time and adjusted a priori for gender, educational level, and IADL disability (at baseline). The exponential of each regression coefficient can be interpreted as a hazard ratio (HR) like in Cox models. All regression coefficients were estimated simultaneously using the Smooth Hazard R package^28^.

Cox proportional hazard models with delayed entry were performed to estimate the risks of hospitalizations, falls, and frailty, using data from the 3C Study. Models were adjusted for gender, educational level, and IADL disability (at baseline).

### Ethics declarations

The Ethical Committee of the University Hospital of Kremlin-Bicêtre (Paris) has approved the protocol of the 3C study. Each participant signed a consent form. The ethical committee of the Bordeaux University Teaching Hospital has approved the PAQUID study according to the principles embodied in the declaration of Helsinki in1988. The study investigators informed all participants and their proxies of the ongoing research activity. They were free to accept or refuse to participate. Participants provided written informed consent.

## Results

### Characteristics of the 3C study sample

Table 1 shows the baseline characteristics of the 3C sample. The average age of the 2104 participants was 74.6 years old (SD: 5.3), and 61.2% were women. During the 17-year follow-up, 143 (6.8%) participants moved into a senior housing.

**Table 1:** Baseline characteristics of participants of 3C study and characteristics of the residents at the time of entry into senior housing.

Variables	Baseline characteristics of the participants of 3C study		Characteristics of the residents at the time of entry into senior housing	
	n	Mean (SD) or n (%)	N Total	Mean (SD) or N (%)
Age (years)	2104	74.6 (5.1)	143	82.8 (5.9)
Gender (women)	2104	1288 (61.2%)	143	115 (80.4%)
High educational level	2099	1837 (87.5%)	143	119 (83.2%)
MMSE score	2081	27.2 (2.4)	139	25.9 (3.3)
Instrumental Activities of Daily Living (at least > 1IADL)	2095	296 (14.1%)	142	69 (48.6%)
Activities of Daily Living (at least > 1ADL)	2102	30 (1.4%)	143	14 (9.8%)
CESD score	2054	7.5. (7.7)	121	9.8 (8.6)

At the time of admission into a senior housing, the mean age of the 143 participants was 82.8 years old and most of them were women (80.4%). Their mean MMSE score was 25.9 and 48.6% were dependent in at least one IADL (Table 1).

### Effects of moving into a senior housing on the risks of death and nursing home admission

**Figure 1:**
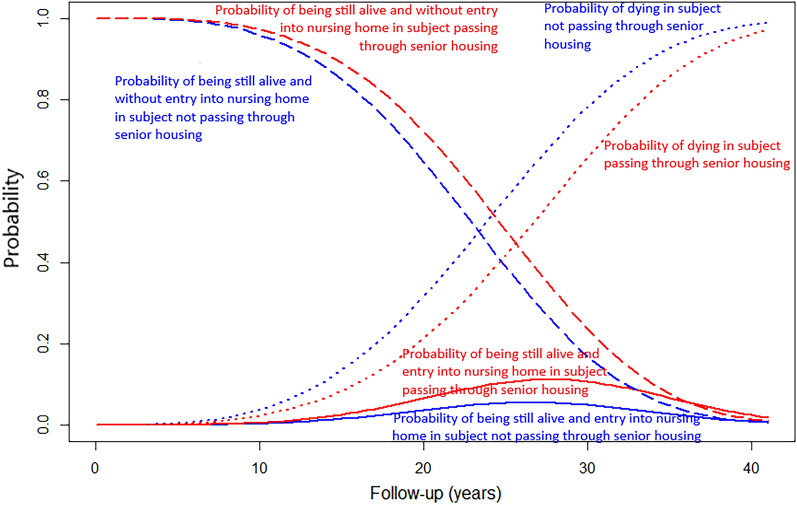
Estimated probabilities of death, nursing home admission, and being alive according to residential status (no senior housing in blue versus senior housing in red).

In all three models (1, univariate; 2, adjusted for sex and education level, and 3, adjusted for sex, education level, and IADL score), moving into a senior housing was associated with a decreased mortality risk over time compared to older adults who did not move into a senior housing (Table 2 and Fig. 1).

**Table 2:** Estimated effects of moving into senior housing on the risks of mortality and nursing home admission, illness-death model. 3C study (n = 2095).

Transition	Model 1 (Univariate)		Model 2 (Adjusted by gende rand education level)		Model 3 (Adjusted by gender, education level and IADL score)	
	HR (CI 95%)	p	HR (CI 95%)	p	HR (CI 95%)	p
Death	0.59 (0.46–0.77)	< 0.001	0.65 (0.50–0.85)	0.001	0.64 (0.49–0.83)	< 0.001
Transition to nursing home	1.63 (1.17–2.27)	0.004	1.58 (1.13–2.20)	0.007	1.54 (1.10–2.15)	0.011
Death after nursing home admission	0.94 (0.63–1.38)	0.739	0.96 (0.65–0.1.43)	0.843	0.96 (0.65–1.43)	0.845

Of the 143 participants who moved into a senior housing during follow-up, 46 (32.2%) subsequently moved into a nursing home. They were 255 (13.0%) among those who never lived in senior housing. In all three models, having lived in a senior housing was associated with an increased risk of nursing home admission (Table 2).

Having lived in senior housing did not affect the risk of death after nursing home entry in all three models (Table 2).

Figure 1 shows the probabilities of death and nursing home admission at each follow-up according to whether the participants moved into a senior housing during follow-up. The probability of dying was higher for older persons who did not move into a senior housing. In contrast, the probability of being still alive without entering a nursing home was higher for those who went through a senior housing.

### Risks of hospitalization, falls and frailty

Among the 143 participants who moved into senior housing, 88 (61.5%) were hospitalized during follow-up, compared to 1389 (70.8%) among those who never moved into senior housing. In all three models, having moved into a senior housing was associated with a decreased risk of hospitalization and falls (Table 3).

**Table 3:** Estimation of the effects of a moving into a senior housing on the risks of hospitalizations, falls, and frailty, Cox model. 3C study (n = 2095).

Variables	Model 1 (Univariate)		Model 2 (Adjusted by gender and education level)		Model 3 (Adjusted by gender, education level and IADL score)	
	HR (CI 95%)	p	HR (CI 95%)	p	HR (CI 95%)	p
Hospitalization	0.52 (0.39–0.70)	< 0.001	0.53 (0.39–0.71)	< 0.001	0.54 (0.40–0.73)	< 0.001
Fall	0.64 (0.51–0.80)	< 0.001	0.64 (0.51–0.80)	< 0.001	0.63 (0.50–0.79)	< 0.001
Frailty	0.60 (0.42–0.86)	0.005	0.65 (0.40–1.06)	0.085	0.63 (0.39–1.05)	0.074

For non-frail older adults, having moved to a senior housing was associated with a decreased risk of frailty incidence only in the univariate model (Table 3). The HR estimates were similar but no more significant when we controlled for gender and educational level.

### Validation analysis in the PAQUID study

#### Characteristics of the PAQUID study sample

Table 1 presents the baseline characteristics of the study sample from the PAQUID study population. The mean age of the 3777 participants was 75.5 years old at baseline (SD: 6.9), and 58.2% of them were women. During the 30-year follow-up, 161 (4.3%) participants moved into a senior housing. At the time of entry into senior housing, the participants were 82.6 (SD: 6.6) years old, predominantly female (78.9%), and 63.8% were dependent in at least one IADL (Table 4). On average, residents remained in senior housing for 3.38 ± 4.55 years.

**Table 4:** Baseline characteristics of participants of PAQUID study and characteristics of the residents at the time of entry into senior housing.

Variables	Baseline characteristics of the participants of PAQUID study		Characteristics of the residents at the time of entry into senior housing	
	n	Mean (SD) or n (%)	N Total	Mean (SD) or N (%)
Age (years)	3777	75.5 (6.9)	161	82.6 (6.6)
Gender (women)	3777	2200 (58.2%)	161	127 (78.9%)
High educational level	3777	2435 (64.5%)	161	93 (57.8%)
MMSE score	3697	25.5 (4.1)	157	24.5 (4.5)
Instrumental Activities of Daily Living (at least > 1IADL)	3770	1148 (30.5%)	160	102 (63.8%)
Activities of Daily Living (at least > 1ADL)	3772	182 (4.8%)	161	18 (11.2%)
CESD score	3628	10.0 (9.2)	149	12.8 (10.7)

#### Effects of moving into a senior housing on the risks of death and nursing home admission

Of the 161 participants who moved into a senior housing during follow-up, 51 (31.7%) subsequently entered a nursing home. In all three models (1, univariate; 2, adjusted for sex and education level; 3, adjusted for sex, education level, and IADL score), moving into a senior housing was associated with a decreased risk of death and an increased risk of nursing home admission (Table 5) compared older adults who did not use senior housing. Having lived in a senior housing did not affect the risk of death once individuals entered a nursing home (Table 5).

**Table 5:** Estimated effects of the move into senior housing on the risks of mortality and nursing home admission, illness-death model. PAQUID cohort (n = 3710).

Transition	Model 1 (Univariate)		Model 2 (Adjusted by gender and education level)		Model 3 (Adjusted by gender, education level and IADL score)	
	HR (CI 95%)	p	HR (CI 95%)	p	HR (CI 95%)	p
Death	0.63 (0.51–0.77)	< 0.001	0.68 (0.56–0.84)	< 0.001	0.72 (0.58–0.88)	0.002
Transition to nursing home	1.46 (1.10–1.94)	0.009	1.34 (1.00–1.78)	0.047	1.39 (1.05–1.86)	0.023
Death after entry in nursing home	0.94 (0.70–1.27)	0.693	0.98 (0.69–1.40)	0.943	1.05 (0.66–1.54)	0.980

## Discussion

With ageing of the population, the demand for long-term services and support for older people is rising. When staying at home becomes difficult (due to deteriorating health, loss of autonomy, absence or exhaustion of caregivers, loneliness, unsuitable housing…), it may be necessary to look for and assess alternatives, such as senior housing or foster families^29^. In these two independent cohorts investigated in this study, we observed that mortality was reduced by about 30% in older adults who had moved into senior housing compared to their counterparts who had not. Moreover, the risk of hospitalization was also reduced by half, as previously described in a study by Park et al.^23^ (even though to a lesser extent). Finally, we observed a decreased risk of falling that is associated with a higher risk of death in the scientific literature^30^.

Previous cohort studies have suggested an increased risk of mortality in senior housing compared with communities in US^20,21^, Australia^19^ or Ireland^22^. Nevertheless, the longitudinal design of our study, with older people initially in their homes, provides a better knowledge on the consequences of entry into senior housing in a life-course perspective than these other short-term studies (1–5 years of follow-up). We noted that the clinical characteristics of older adults living in French senior housing are probably different compared to other countries. In the United States or Canada, the characteristics of older people in senior housing are closer to those of nursing home residents, since a majority of older adults suffer from dementia (58% in the Canadian study by Maxwell et al*.*^31^ or 68% of individuals in the American study by Watson et al*.*^32^). In our cohorts, the mean age of older adults entering a senior housing was 82.8 years at admission and the mean MMSE score was 25.2 ± 3.3. Therefore, whereas senior housing is often considered in other countries as an alternative to nursing homes, our results suggest that senior housing in France is rather a stepping-stone between home care and nursing home care. Indeed, compared to older adults who did not move into a senior housing, the health trajectory of older adults who lived at home and then moved to a senior housing puts them at increased risk of nursing home admission. Indeed, about 30% of the residents of senior housing moved to a nursing home, likely due to cognitive or functional decline.

In France, the target population for senior housing is rather older people who are rather frail (or at risk of frailty) and for whom their housing is no longer suitable^17^. A senior housing offers many potential advantages: safe housing (no stairs, adapted lightnings, especially at night, bathrooms and toilets designed to avoid the risk of falling), personal support, and health care, along with the facilitation of social interactions. Senior housing could promote autonomy of older adults—the primary concern in this population—and help manage the health trajectory of vulnerable older people. We have observed that senior housing reduce mortality risks, often the primary concerns of families and health professionals^33^. For practical and logistical reasons, senior housing may be a more appropriate setting for secondary prevention than the general population, where frail older people are scattered. The array of medical and support services in senior housing may help adapt to the constraints and needs of older adults to promote lifestyle-based group interventions such as diet, physical activity, and social interactions^34^. Finally, staffing levels in these facilities, particularly nursing staff, are thought to play a pivotal role in preventing adverse health events. Stearns et al.^35^ found an association between reduced rates of hospital admissions and a higher number of licensed staff in senior housing.

In this study, several limitations are noteworthy. The sample size was small for older adults who used senior housing. Nevertheless, the results are consistent across the two independent cohorts we investigated. The demographic characteristics of the participants were similar to those of a recent French cohort living in senior housing^18^. As stated above, the clinical characteristics of older people in the French senior housing are different from those observed in other countries, which limits comparisons with the scientific literature. We hypothesize that housing security, improved care, and support management may explain the lower mortality observed in the senior housing group. Nevertheless, other factors could have been studied, such as medication use, marital status or cognitive decline^36^. Moreover, our cohorts do not include systematically collected information on patient characteristics, such as previous hospitalizations and comorbidities, which are often the main causes of admission into an institution and are significant predictors of mortality. Further research would be necessary to understand the most appropriate profile of senior housing residents, mainly in terms of cognitive functioning, and to determine the factors leading to nursing home admission in people living in senior housing^36^. This transition may happen too late, as senior housing and nursing homes still suffer from a pejorative representation in the general population. Finally, from a public health perspective, medico-economic studies should assess the cost-efficiency of senior housing.

## Conclusion

Our results support the view that senior housing as appropriate transitional care option for older people when living at home becomes challenging. In the two independent cohorts investigated in this study, we observed lower risks of mortality and hospitalization in older adults who moved into senior housing compared to their counterparts who did not. We need to know more of the benefits of these facilities and the characteristics of older adults who might benefit most from them.

## Data Availability

The datasets used and/or analysed during the current study are available from the corresponding author on reasonable request.
